# Natural Products of Pharmacology and Mechanisms in Nucleus Pulposus Cells and Intervertebral Disc Degeneration

**DOI:** 10.1155/2021/9963677

**Published:** 2021-08-03

**Authors:** Hai-Wei Chen, Guang-Zhi Zhang, Ming-Qiang Liu, Li-Juan Zhang, Ji-He Kang, Zhao-Heng Wang, Wen-Zhao Liu, Ai-Xin Lin, Xue-Wen Kang

**Affiliations:** ^1^Department of Orthopedics, Lanzhou University Second Hospital, Lanzhou, Gansu 730000, China; ^2^The Second Clinical Medical College, Lanzhou University, Lanzhou, Gansu 730000, China; ^3^Department of Endocrinology, Lanzhou University Second Hospital, Lanzhou, Gansu 730000, China; ^4^Key Laboratory of Orthopedics Disease of Gansu Province, Lanzhou University Second Hospital, Lanzhou, Gansu Province 730030, China; ^5^The International Cooperation Base of Gansu Province for The Pain Research in Spinal Disorders, Lanzhou, Gansu Province 730030, China

## Abstract

Intervertebral disc degeneration (IDD) is one of the main causes of low back pain (LBP), which severely reduces the quality of life and imposes a heavy financial burden on the families of affected individuals. Current research suggests that IDD is a complex cell-mediated process. Inflammation, oxidative stress, mitochondrial dysfunction, abnormal mechanical load, telomere shortening, DNA damage, and nutrient deprivation contribute to intervertebral disc cell senescence and changes in matrix metabolism, ultimately causing IDD. Natural products are widespread, structurally diverse, afford unique advantages, and exhibit great potential in terms of IDD treatment. In recent years, increasing numbers of natural ingredients have been shown to inhibit the degeneration of nucleus pulposus cells through various modes of action. Here, we review the pharmacological effects of natural products on nucleus pulposus cells and the mechanisms involved. An improved understanding of how natural products target signalling pathways will aid the development of anti-IDD drugs. This review focuses on potential IDD drugs.

## 1. Introduction

Low back pain is a symptom of intervertebral disc degeneration (IDD). Approximately 70% to 85% of people will experience low back pain or neck pain at least once in their lives, which severely reduces their quality of life [1]. Dieleman et al. reported that, among the populations with the highest medical expenditures from 1996 to 2016, patients with low back pain, neck pain, and diabetes comprised the greatest proportions. Moreover, the total cost of back pain, neck pain, and other musculoskeletal diseases is $264.3 billion, which constitutes a considerable economic burden both to society and to the families of affected individuals [2]. IDD is mainly characterised by the degradation of extracellular matrix (ECM) and the loss of nucleus pulposus (NP) cells [3]. However, the specific pathogenesis of IDD is not fully understood. Current research indicates that IDD is a complex process that ultimately leads to changes in cell function and structure [4]. Notably, inflammation, oxidative stress, mitochondrial dysfunction, abnormal mechanical load, telomere shortening, DNA damage, and nutrient deprivation are involved in intervertebral disc cell senescence and altered matrix metabolism, which ultimately lead to the development of IDD [5].

Currently, therapeutic approaches for patients with IDD symptoms include conservative treatment and surgical intervention, although there is no cure or established method of disease management [6]. Therefore, safe and effective IDD treatment methods require further exploration. Mesenchymal stem cells [7], tissue engineering technology [8], growth factor therapy [9], exosomes [10], and biomaterial therapy [11] are currently the most compelling research avenues for IDD treatment. Although these approaches are novel and have great potential for further development, their clinical applications remain challenging.

Natural products are derived from plants, animals, and microorganisms [12]. Natural products of plant origin exhibit powerful pharmacological activities. *Ophiorrhiza rugosa* leaves reduce chemical and heat-induced inflammation [13]. Spirulina (a “superfood”) exhibits great potential for treatment of inflammatory diseases [14]. The structural diversity of natural products is associated with many different pharmacological activities and functions; the materials interact with various proteins and other biological molecules [15–17]. There has been increasing attention towards the pharmacological effects of natural products on NP cells and the mechanisms involved. Many plant compounds inhibit NP cell senescence and apoptosis [18, 19]. Here, we review these findings and offer suggestions for the development of anti-IDD drugs.

## 2. Pathophysiology of IDD

In healthy individuals, the intervertebral disc is a fibrocartilage structure consisting of three parts: the central NP, peripheral annulus fibrosus, and cartilage endplate (CEP) on both sides [20, 21]. The NP is mainly composed of ECM rich in type II collagen, elastin, and proteoglycan. This area mainly acts to offset and transmit axial pressure load during stress to the spine. In contrast, the ECM of annulus fibrosus cells is mainly composed of alternating type I collagen fibres. Their main function is to prevent the NP from protruding under pressure during bending or twisting of the spine. Finally, the CEP is hyaline cartilage tissue with uniform thickness, and its ECM is mainly composed of proteoglycans and collagen fibres [22–24]. Because the intervertebral disc is an avascular and non-nervous tissue, the CEP plays an important role in nutrient supply to the intervertebral disc. Most nutrient exchanges and metabolic wastes occur through the CEP to maintain normal intervertebral disc structure and function [22]. As IDD progresses, the local metabolic status changes for intervertebral disc NP cells, annulus fibrosus cells, and CEP cells. These alterations involve reduced anabolism and enhanced catabolism, which lead to greater ECM degradation, diminished NP water content, lowered annulus fibrosus elasticity, and increased CEP calcification. These changes ultimately lead to pathological changes such as poor spinal stability, intervertebral disc collapse, spondylolisthesis, osteophyte formation, annulus fibrosus tearing, and NP tissue protrusion, all of which combine to cause low back pain [25–27]. The main causes of IDD are described below.

### 2.1. Inflammation

Inflammation is a pathological process that occurs after infection or injury. During inflammation, cells can secrete various inflammatory factors (e.g., TNF-*α*, IL-1*α*/*β*, IL-6, IL-8, IL-2, IL-17, IL-10, IL-4, IFN-*γ*, and PGE2). These factors become involved in a series of inflammatory reactions that promote ECM degradation, autophagy, aging, and intervertebral disc cell apoptosis, thereby causing intervertebral disc dysfunction and structural changes [6, 28, 29].

### 2.2. Oxidative Stress

Cellular aerobic metabolism is an important component of cell structure and function, and reactive oxygen species (ROS) are the main metabolic by-products. With the development of IDD, the weakened antioxidant capacity in the intervertebral disc causes ROS aggregation. This leads to oxidative stress and activation of various IDD signalling pathways, which accelerate cell apoptosis, senescence, and ECM catabolism [30, 31].

### 2.3. Mitochondrial Dysfunction

Mitophagy eliminates excessive ROS to maintain mitochondrial homeostasis, but mitochondrial DNA damage generates excessive stress signals and increases the ROS content. Excessive ROS levels eventually lead to mitochondrial dysfunction, trigger programmed cell death, and accelerate the progression of IDD [32–34].

### 2.4. Abnormal Mechanical Load

In a healthy intervertebral disc, appropriate biomechanical stress and hydrostatic pressure are necessary to maintain intervertebral disc homeostasis. When they are mechanically overloaded, cells within the intervertebral disc stop producing proteoglycans, thereby reducing pressure in the intervertebral disc and increasing the shearing force in the NP. This greater shear force further reduces proteoglycan production, forming a vicious circle that accelerates IDD [35, 36].

### 2.5. Telomere Shortening and DNA Damage

During continuous cell growth and division, incomplete replication of DNA ends and reduced telomerase activity leads to shortened telomere length and changes in DNA structure. Additional DNA damage due to exogenous factors can activate cell senescence mechanisms and lead to intervertebral disc cell rigidity [5, 37].

### 2.6. Nutritional Deprivation

Intervertebral discs are tissues without blood vessels and nerves. Most intervertebral disc nutrients and metabolic wastes are exchanged by diffusion through the CEP. A reduction in the nutrient supply leads to a diminished oxygen level and elevated lactic acid concentration, which changes pH, affects cell function, and alters ECM synthesis [5, 22, 38].

## 3. Natural Pharmacological Products

Natural products are mainly extracted from various herbs. These products are structurally divided into six categories: phenolics, flavonoids, alkaloids, terpenoids, saponins, and quinones. They regulate oxidative stress, inflammation, apoptosis, and matrix metabolism through different mechanisms. Oxidative stress compromises NP cell function [31]. Natural products can downregulate mitochondrial ROS levels, increase SOD activity and the expression of mitochondrial cytochrome c, and protect cells from oxidative stress. Inflammation also compromises NP cell activity [39]. Natural products inhibit the expression of inflammatory factors IL-1*β*, TNF-*α*, COX-2, iNOS, PGE2, and IL-6, thereby reducing cell damage caused by inflammation. Apoptosis (programmed cell death) is a key regulator of NP cell activities [40]. The proapoptotic proteins include Bax, caspase-3, and caspase-9; antiapoptotic proteins include Bcl-2. The ECM maintains intervertebral disc function and stability; a disintegrin, a metalloproteinase with a thrombospondin motif (ADAMTS), and other matrix metalloproteinases (MMPs) mediate both ECM degradation and synthesis [41]. Natural products inhibit the expression of MMP1, MMP3, MMP13, ADAMTS-4, and ADAMTS-5, thereby promoting collagen II and aggrecan synthesis. Here, we briefly introduce the pharmacological effects and molecular mechanisms of these six types of natural compounds in the treatment of IDD.

### 3.1. Phenolics

#### 3.1.1. Resveratrol

Resveratrol (RES) is a naturally occurring polyphenol compound that exists in various plants and is mainly extracted from cinnamon bark, gourd, and polygonum cuspidatum. It has antioxidant, anti-inflammatory, antiaging, and autophagy-regulating effects, as well as the ability to reduce mechanical pain [42, 43]. Wang et al. [18] reported that RES activates the Nrf2/Heme oxygenase 1 signalling pathway in rat NP cells, reducing ROS levels, slowing NP cell senescence, and enhancing NP cell matrix anabolism. Jiang et al. [44, 45] found that RES activates the PI3K/Akt pathway to inhibit cell apoptosis during IL-1*β*-mediated inflammation. RES has been shown to reduce mechanical overload damage in a dose-dependent manner and regulate NP cell apoptosis by inhibiting activation of the ERK1/2 pathway [46]. It also inhibits the ROS/NF-*κ*B signalling pathway and slows NP degeneration [47]. RES can upregulate SIRT1 (a member of the NAD^+^-dependent histone deacetylase family, involved in age-related diseases, cancers, and degenerative diseases [34]), which inhibits the p21/p16 pathway and regulates NP cell senescence [48]. In addition, RES can activate autophagy through the AMPK/SIRT1 axis and control cell matrix metabolism [49]. RES upregulates the expression of SIRT1 and MMP1, thus increasing the synthesis of the NP cell matrix [50, 51].

Notably, RES can also improve the degree of IDD by acting on the Akt-FoxO1-SIRT1 axis and activating the mTOR/caspase-3 signalling pathway [52, 53]. Therefore, it has potential efficacy as treatment for IDD. At present, RES is presumed to control inflammation and oxidative stress, regulate autophagy, and reduce mechanical load damage. However, the cytotoxic and genotoxic effects of RES have not been elucidated, which has led to substantial challenges in terms of clinical applications. Further research is needed concerning the clinical applications of RES in IDD treatment, as well as the prevention of its side effects.

#### 3.1.2. Coumarin

Coumarin is a natural phenolic compound from *Solanaceae*, *Rutaceae*, and *Amburana cearensis* [54, 55]. It has anti-inflammatory and analgesic functions [56, 57]. Coumarin scavenges active oxygen moieties, reducing oxidative stress; it also controls multiple signalling pathways [58, 59]. Su et al. [19] reported that isofraxidin, a coumarin compound, inhibits the NP inflammatory factors COX-2, iNOS, PGE2, TNF-*α*, IL-6, MMP3, and MMP13; the expression of these factors is induced by IL-1*β*. Isofraxidin also enhances the expression of collagen II and aggrecan through the NF-*κ*B signalling pathway. Acid-sensitive ion channel 3 is a pH sensor, mainly expressed in nociceptors [60]. He et al. [61] demonstrated that osthole, a natural coumarin derivative, reduces the expression of acid-sensitive ion channel 3 in the dorsal root ganglia and mitigates NP-related mechanical pain. Sparstolonin B is also termed isocoumarin. Ge et al. [62] found that sparstolonin B alleviated inflammation, oxidative stress, and apoptosis of traumatised rat NP cells by inhibiting TLR4/NF-*κ*B signalling or activating PI3K/Akt signalling.

Although there have been relatively few studies on coumarin in IDD, we expect that coumarin has great potential in IDD research, based on its anti-inflammatory, antioxidant, and analgesic properties. Therefore, investigation of the molecular mechanism of coumarin is important for its future application in the treatment of IDD.

#### 3.1.3. Curcumin and o-Vanillin

Curcumin (CUR) is a lipophilic polyphenolic substance, mainly isolated from the rhizome of the herbaceous plant turmeric [63]. It has anti-inflammatory and antioxidant properties, promotes cell proliferation, and regulates senescence and autophagy signalling pathways [64, 65]. CUR is actively used in the treatment of aging-related diseases, including cardiovascular diseases, atherosclerosis, neurodegenerative diseases, rheumatoid arthritis, osteoporosis, diabetes, hypertension, chronic kidney disease, and chronic inflammatory diseases [66, 67]. In the context of IDD, Kang et al. [68] found that CUR can activate the AMPK/mTOR/ULK1 signalling pathway to enhance autophagy. This reduces ROS production; increases cytochrome c production; and protects NP cells from apoptosis, senescence, and ECM degradation caused by oxidative stress. o-Vanillin is the principal metabolite of curcumin. Cherif et al. [69] reported that curcumin and o-vanillin reduced the number of SA-*β*-gal-positive cells, thus slowing NP cell aging; lowered the expression levels of the inflammatory cytokines IL-6, IL-8, MMP3, and MMP13; and increased the levels of collagen II and aggrecan. These effects were mediated by the Nrf2 and NF-*κ*B pathways. Cherif et al. [70] showed that o-vanillin upregulated CDK6, CDK2C, CDC25C, and other cell cycle genes, while it downregulated many common SASP factors including INF-*γ* and IL-6; these changes led to slower human NP cell senescence.

Nevertheless, there have been few studies on CUR in IDD, and its underlying mechanism is not fully understood. However, based on its abundance and unique biological activity, the potential molecular mechanism of CUR should be explored in the treatment of IDD to benefit patients with IDD.

#### 3.1.4. Honokiol

Honokiol (HKL) is a natural bisphenol compound, mainly isolated from the leaves and bark of Magnolia plants [71]. It has anti-inflammatory, antioxidant, antiaging, and antiapoptosis properties, as well as the ability to regulate mitochondrial function and autophagy signalling pathways [72, 73]. Tang et al. [74] reported that HKL inhibits the activation of NF-*κ*B, which reduces the production of ROS in NP cells and the expression of inflammatory factors COX-2, iNOS, IL-1*β*, and IL-6. It also regulates apoptosis and matrix metabolism in NP cells. Notably, SIRT3 is a member of the NAD^+^-dependent histone deacetylase family that regulates the activities of key oxidative phosphorylation enzymes through deacetylation, thereby controlling mitochondrial energy metabolism [75]. Wang et al. [76] reported that HKL upregulated the expression of SIRT3 in NP cells through the AMPK/PGC-1*α* signalling pathway. Moreover, SIRT3 can downregulate the level of O^2−^, while upregulating the activity of SOD to maintain mitochondrial function and promote autophagy; these effects inhibit apoptosis and senescence in NP cells. Animal experiments have shown that HKL has a therapeutic effect on IDD. Chen et al. [77] used high-performance liquid chromatography to explore the distribution of HKL in rat intervertebral discs. In vitro analysis showed that HKL diffusion into the discs was concentration-dependent. HKL is of plant origin and thus exhibits low toxicity and few side effects; HKL slowed IDD progression when injected into animals. Therefore, HKL may be useful in the treatment of human IDD.

#### 3.1.5. Salvianolic Acid

Salvianolic acid is the most abundant polyphenolic of *Salvia miltiorrhiza*; it exhibits anti-inflammatory, anticancer, antioxidant, and cardioprotective effects [78]. Dai et al. [79] found that salvianolic acid B activated the JAK2/STAT3 signalling pathway, reduced ROS and MDA production, and upregulated GSH and SOD2 production to maintain mitochondrial function in NP cells. Transformation of the mesenchymal stem cells (MSCs) of IDD patients has attracted considerable attention. Yan et al. [80] found that salvianolic acid B increased MSC collagen II and aggrecan production, indicating that the material promoted MSC differentiation into NP cells. Although there have been few relevant studies, we believe that salvianolic acid B has great potential in terms of IDD treatment; the material promotes MSC differentiation into NP cells, which may greatly aid IDD patients.

#### 3.1.6. Other Phenolics

Green tea polyphenols exhibit antioxidant and anti-inflammatory effects [81]. NOX activity is the principal source of extra-mitochondrial ROS. Song et al. [82] showed that tea polyphenols significantly inhibited the production of iNOS, NOX4, and ROS, as well as MMP3 expression, in NP cells treated with H_2_O_2_. Polyphenols increased the levels of collagen II, aggrecan, and SOX-9 by regulating the KEAP1/NRF2/ARE pathway. Pigallocatechin-3-gallate is another natural polyphenol of green tea [83]. Tian et al. [84] reported that IDD was associated with upregulation of cGAS, STING, and NLRP3; moreover, pigallocatechin-3-gallate inhibited the cGAS/STING/NLRP3 pathway and exerted antiapoptotic and anti-inflammatory effects. Gallic acid is a natural phenolic (a secondary metabolite of grape seeds, *Syzygium fruticosum* fruit, and other plants [85]). Gallic acid exerts strong antioxidant and anti-inflammatory effects [86]. Huang et al. [87] found that gallic acid inhibited TNF-*α*-induced apoptosis and ADAMTS-4 production in NP cells by regulating NF-*κ*B signalling. Sesamin is a natural polyphenol of sesame seeds that inhibits inflammation, proliferation, apoptosis, and other cellular actions [88]. Li et al. [89] found that sesamin reduced matrix catabolic enzyme expression (MMP1, MMP3, MMP13, ADAMTS-4, and ADAMTS-5) and the levels of the inflammatory factors IL-1*β*, TNF-*α*, iNOS, COX-2, and PGE2 in rat NP cells by inhibiting the MAPK pathway.

**Figure 1:**
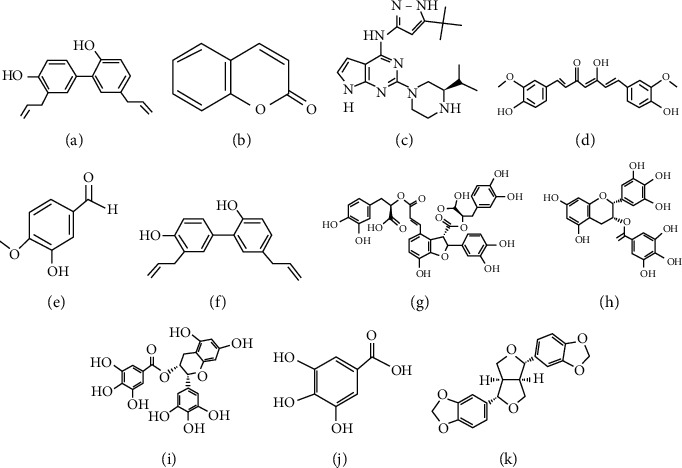
Chemical structures of phenolics with pharmacological potential: (a) resveratrol; (b) coumarin; (c) sparstolonin B; (d) curcumin; (e) o-vanillin; (f) honokiol; (g) salvianolic acid B; (h) tea polyphenol; (i) pigallocatechin-3-gallate; (j) gallic acid; (k) sesamin.

Polyphenols (also termed polyhydroxyphenols) are widespread in herbal medicines, tea, coffee, grains, and vegetables. They control inflammation and oxidative stress, while regulating autophagy; thus, they delay IDD progression and promote MSC differentiation into NP cells. The multiple pharmacological effects and multitargeting properties of natural polyphenols show great potential. However, further evidence of their utilities as IDD treatments is required (Table 1 and Figure 1).

### 3.2. Flavonoids

#### 3.2.1. Icariin

Icariin (ICA) is a prenyl flavonoid and the main biologically active substance of herbal medicines [90]. It has antioxidant and anti-inflammatory functions, and it can maintain the integrity of mitochondrial membranes [91]. ICA can regulate the activities of telomeres, telomerase, and sirtuin proteins, as well as energy metabolism pathways and cell senescence [92]. Hua et al. [93] showed that ICA can inhibit the production of ROS and increase the expression of cytochrome c, which reduces oxidative stress damage to NP cells and inhibits apoptosis through the Nrf2 signalling pathway. Hua et al. [94] reported that ICA reduces the expression of COX-2 and iNOS in NP cells, inhibits the production of matrix-degrading enzymes, and enhances matrix anabolism by inhibiting the MAPK and NF-*κ*B signalling pathways. In an in vitro experiment, Deng et al. [95] demonstrated that ICA can reduce the levels of ROS, caspase-3, and Bax in NP cells; it can also inhibit cell apoptosis by activating the PI3K/Akt signalling pathway. ICA may improve the viability and function of cryopreserved human NP mesenchymal stem cells in addition to improving cell adhesion and maintaining mitochondrial function [92].

Although ICA has shown great potential in the treatment of IDD, its research in the context of IDD is relatively limited. In particular, its therapeutic effects and underlying molecular mechanisms require further analysis. Although ICA can slow cell senescence by regulating the activities of telomeres, telomerase, and sirtuin proteins, there is insufficient experimental evidence that ICA can slow the development of IDD. Therefore, further experimental verification is needed.

#### 3.2.2. Naringin

Naringin (NRG) is a natural flavonoid compound, mainly extracted from citrus fruits [96]. It has anti-inflammatory, antioxidant, and antiapoptotic properties; it can regulate both autophagy and gluconeogenesis [97]. In addition, NRG can regulate molecular targets such as HMG-CoA reductase, NF-*κ*B, AMPK, Nrf2, and ROS [98]. NRG exhibits anti-bone resorption and antifat effects, enhances bone features, improves the bone microenvironment, and helps reconstruct bone structure [99]. Li et al. [100] showed that NRG can increase human NP cell viability and inhibit the expression of inflammatory cytokine TNF-*α*, thereby enhancing the expression of collagen II and aggrecan. Zhang et al. [101] reported that, under TBHP-mediated oxidative stress, NRG promotes autophagy in rat NP cells by activating the AMPK signalling pathway, which inhibits cell apoptosis and increases the production of collagen II and aggrecan. Gao et al. [102] found that NRG can substantially reduce the expression of inflammatory cytokines TNF-*α* and IL-6; it can improve the matrix anabolic ability of NP cells through the NF-*κ*B signalling pathway via IL-1*β*-mediated induction. Nan et al. [103] demonstrated that NRG inhibits the level of ROS and increases mitochondrial membrane potential in rat NP-derived mesenchymal stem cells, which improves mitochondrial function and reduces the apoptosis of NP mesenchymal stem cells through the PI3K/Akt signalling pathway. Current research regarding the use of NRG in IDD is limited and its effectiveness has not been confirmed, although its antioxidant, antiapoptotic, anti-inflammatory, and autophagy-regulating effects can protect NP cells. Moreover, NRG can regulate gluconeogenesis. Further research is needed to support the use of NRG in clinical treatment of IDD, including qualitative and quantitative analyses of the mechanism underlying IDD and assessment of NRG efficacy in the treatment of IDD.

#### 3.2.3. Genistein

Genistein (GES) is a natural isoflavone compound, mainly extracted from soybeans [104]. It has anti-inflammatory, antioxidant, and antiapoptotic properties, as well as the ability to improve mitochondrial function [105]. Heme oxygenase 1 and quino oxidase 1 play important roles in the Nrf2-mediated antioxidant defence system [106]. Wang et al. [107] studied the GES-mediated activation of the Nrf2 signalling pathway and found reduced levels of ROS in NP cells, which inhibited cell apoptosis and ECM catabolism. Animal experiments also confirmed this conclusion. Ge et al. [108] demonstrated that GES has protein tyrosine kinase inhibitory function, such that it blocks activation of the p38 MAPK pathway and reduces the expression of inflammatory factors TNF-*α* and IL-1*β* in rat NP cells, thereby promoting the expression of collagen II and aggrecan. The therapeutic effects of GES were confirmed in animal experiments. The current knowledge concerning GES is relatively limited; therefore, the abilities of GES to impact IDD through anti-inflammatory, antioxidant, and antiapoptotic properties require further research studies.

#### 3.2.4. Wogonin

Wogonin (WG) is a natural flavonoid compound extracted from the root extract of *Scutellaria baicalensis* Georgi [109]. It has antioxidant and anti-inflammatory activities [110]. Fang et al. [111] reported that WG can inhibit the expression of inflammatory factors COX-2 and iNOS, as well as the activities of MMP3, MMP13, and ADAMTS-4. Moreover, it can promote the expression of collagen II and aggrecan, which are regulated by the Nrf2/ARE signal axis. Although there has been minimal research concerning WG, the Nrf2/ARE signalling pathway is a well-known pathway involved in IDD molecular signalling. Thus, WG may have potential treatment applications in IDD, but supporting experimental data are needed.

#### 3.2.5. Luteoloside

Luteoloside (Lut) is a natural flavonoid of honeysuckle, lettuce, silver flower, and salvia [112]. Lut exerts antioxidant, anti-inflammatory, antiautophagy, and analgesic effects [113]. Lin et al. [114] demonstrated that Lut inhibited NF-*κ*B signalling and the expression of the inflammatory factors iNOS, COX-2, PGE2, NO, TNF-*α*, and IL-6 in rat NP cells. Lut also regulated the expression of key molecules involved in matrix degradation (MMP13 and ADAMTS-5), thereby inhibiting apoptosis and promoting the production of collagen II and aggrecan. Animal experiments have confirmed the therapeutic effects of Lut on IDD. Although few human studies have appeared, the anti-inflammatory and antioxidant properties suggest that further research is desirable.

#### 3.2.6. Quercetin

Quercetin is a naturally occurring phenolic of vegetables, fruits, and *Gynura* plants that may protect against a number of diseases of aging including osteoporosis and heart disease [115, 116]. Wang et al. [117] showed that quercetin activated the SIRT1 autophagy signalling pathway and reduced TBHP-induced ROS production, thereby inhibiting NP cell apoptosis and matrix catabolism. Animal experiments confirmed the therapeutic effects of quercetin on IDD. Shao et al. [118] reported that quercetin may bind to the KEAP1-NRF2 complex to inhibit the NF-*κ*B pathway, thus reducing the IL-1*β*-mediated expression of IL-6, IL-8, MMP13, and MMP3; this alleviates NP cell senescence. There is minimal evidence that quercetin can slow IDD development; more work is needed.

#### 3.2.7. Other Flavonoids

Baicalein is a natural flavonoid from the roots of *Scutellaria baicalensis* and *Scutellaria lateriflora*, which exhibits antioxidant and anti-inflammatory activities [119]. Jin et al. [120] found that baicalein inhibited IL-1*β* production by NP cells and increased NP ECM synthesis by regulating NF-*κ*B and MAPK signalling. Apigenin is a flavonoid of Asteraceae [121]. Ding and Li [122] found that apigenin ameliorated inflammatory factor expression and inhibited cell matrix metalloprotein synthesis by acting on the TNF-*α* signalling pathway. Kaempferol is a flavonoid of *Ginkgo* and *Moringa* that exhibits antiaging, antioxidant, anti-inflammatory, and antiosteoporosis effects [123]. Bone marrow-derived mesenchymal stem cells represent a potential autologous stem cell source for NP regeneration. Zhu et al. [124] found that, by activating NF-*κ*B, kaempferol reduced the levels of the proinflammatory cytokine IL-6 in bone marrow-derived mesenchymal stem cells; it also enhanced SOX-9, collagen II, and aggrecan production by those cells.

**Figure 2:**
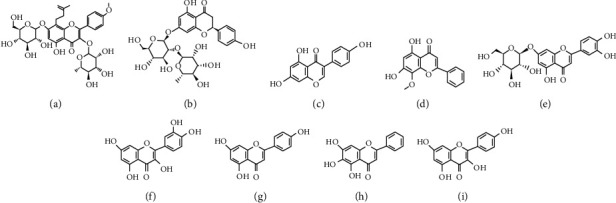
Chemical structures of phenolics with pharmacological potential: (a) icariin; (b) naringin; (c) genistein; (d) wogonin; (e) luteoloside; (f) quercetin; (g) baicalein; (h) apigenin; (i) kaempferol.

Flavonoids are widespread in fruits, vegetables, herbs, and other plant foods. The activities of hydroxyphenolic flavonoids are structure-dependent. Natural flavonoids exert anti-inflammatory and antioxidant effects; they slow cell senescence by regulating senescence-associated enzymes. Such products may be useful in the treatment of IDD, but more data are needed (Table 2 and Figure 2).

### 3.3. Alkaloids

#### 3.3.1. Berberine

Berberine (BBR) is a quaternary ammonium alkaloid isolated from the traditional Chinese medicine Rhizoma coptidis, which has antioxidant, anti-inflammatory, and antiapoptotic effects [125]. BBR and its derivatives, as well as associated pharmaceutical preparations, have good effects on cancer, obesity, diabetes, inflammation, atherosclerosis, Alzheimer's disease, rheumatoid arthritis, and cardiovascular diseases [126]. Lu et al. [127] reported that BBR inhibits the activation of NF-*κ*B signalling and the expression of caspase-3, MMP3/13, and ADAMTS-4/5, thereby exerting antiapoptotic and ECM catabolism effects. Luo et al. [128] demonstrated that BBR reduced the production of ROS in NP cells and inhibited cell apoptosis by regulating both endoplasmic reticulum stress and autophagy through the IRE1/JNK pathway. Chen et al. [129] studied the therapeutic effect of BBR in the treatment of IDD, which may reduce NP cell apoptosis and inhibit ECM catabolism by activating autophagy.

#### 3.3.2. Oxymatrine

Oxymatrine is a quinazine alkaloid from *Sophora flavescens* that reduces inflammation, oxidative stress, and apoptosis [130]. Wei et al. [131] reported that, by regulating NF-*κ*B signalling, oxymatrine reduced MMP levels in NP cells; it also reduced IL-1*β*-induced apoptosis of those cells. Furthermore, oxymatrine increased collagen II and aggrecan expression in NP cells. Animal experiments confirmed these protective effects. Liposomes are biocompatible nanocarriers used to deliver various drugs. Wang et al. [132] found that such delivery enhanced drug distributions in targeted areas. Oxymatrine-liposomes enhanced drug accumulation in intervertebral discs, while reducing NP cell apoptosis and ECM degeneration.

#### 3.3.3. Other Alkaloids

Piperine is an alkaloid of *Piper longum* L. [133] that exhibits antioxidant, antisenescence, and immunomodulatory effects [134]. Li et al. [135] demonstrated that piperine inhibited LPS-mediated JNK phosphorylation and NF-*κ*B activation, thus reducing the levels of the inflammatory factors IL-1*β*, TNF-*α*, IL-6, and iNOS; piperine also enhanced matrix anabolism in NP cells.

**Figure 3:**
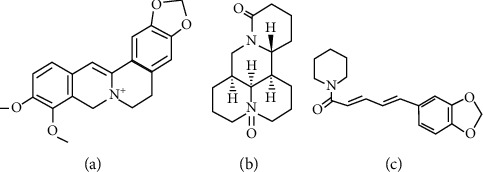
Chemical structures of alkaloids with pharmacological potential: (a) berberine; (b) oxymatrine; (c) piperine.

Our current knowledge of alkaloids is relatively limited. Determination of the capacities of alkaloids to inhibit IDD requires further research; the liposomal combination approach may offer a new path towards IDD treatment (Table 3 and Figure 3).

### 3.4. Terpenoids

#### 3.4.1. Andrographolide

Andrographolide is a natural terpenoid of the Acanthaceae family, commonly termed “King of the bitters” or “Kalmegh” [136], that exerts antioxidant, anti-inflammatory, and antiapoptotic effects [137]. Liu et al. [138] showed that andrographolide inhibited production of the inflammatory factors COX-2 and PGE2, while reversing MMP3, MMP13, ADAMTS-4, and ADAMTS-5 overexpression, by inhibiting NF-*κ*B activation. Zhang et al. [139] reported that andrographolide inhibited the expression of several matrix metalloproteinases (MMP3, MMP9, and MMP13), as well as apoptosis, by controlling NF-*κ*B signalling.

#### 3.4.2. Other Terpenoids

Glycyrrhizic acid is a triterpene from liquorice roots and rhizomes that exerts anti-inflammatory and antioxidant effects [140]. Liu et al. [141] found that glycyrrhizic acid inhibited the expression of the inflammatory factors TNF-*α*, IL-6, IL-8, and iNOS, as well as apoptosis, by reducing p38/p-JNK signalling; thus, it prevented NP cell degradation. CDDO and its derivatives are terpenoids derived from oleanolic acid that exert anti-inflammatory, anticancer, and antioxidant effects. Zhang et al. [142] found that Nrf2 activation was essential for the overexpression of HO-1 induced by CDDO-ethyl amide (EA); this protected NP cells from the oxidative stress induced by high glucose. Cannabinoids are natural terpenoids from hemp [143] that exhibit anti-inflammatory and antimicrobial effects [144]. Cannabidiol was injected into rat intervertebral discs injured via needle puncture. MRI and histological analysis revealed that cannabidiol significantly reduced intervertebral disc damage [145].

**Figure 4:**
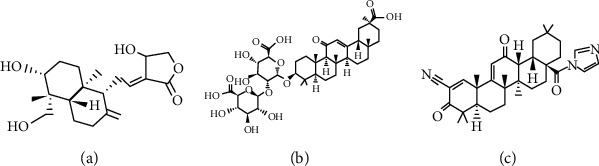
Chemical structures of terpenoids with pharmacological potential: (a) andrographolide; (b) glycyrrhizin; (c) CDDO-EA.

The anti-inflammatory and antioxidant terpenoids enhance the repair cascade at sites of intervertebral disc injury. They are expected to restore NP cell function. Future analyses of the molecular mechanisms involved and consideration of appropriate administration routes are important (Table 4 and Figure 4).

### 3.5. Saponins and Quinones

#### 3.5.1. Ginsenosides

Ginsenosides (GSs) are saponins, also termed triterpene saponins, mainly found in medicinal materials from the ginseng genus [146]. According to the position and quantity of the sugar moiety in ginseng-derived sugar, GSs are divided into three types: protopanaxadiol, protopanaxatriol, and oleanolic acid, all of which have anti-inflammatory, antioxidant, and antiapoptotic effects [147]. Yu et al. [148] showed that the GSs Rg1 regulates Wnt/*β*-catenin signalling, promotes the proliferation of rat NP cells and the production of collagen II and aggrecan, and reduces cell apoptosis. Chen et al. [149] reported that the GSs Rg3 inhibits the NF-*κ*B signalling pathway and reduces the production of ROS in NP cells, thereby promoting cell proliferation and ECM anabolism, while reducing apoptosis.

#### 3.5.2. Plumbagin

Plumbagin is a quinone from the root of the medicinal herb “graphite” that exhibits anti-inflammatory and antioxidant effects [150]. Chu et al. [151] found that plumbagin reduced NP cell oxidative stress and inflammation induced by H_2_O_2_ by modulating NF-*κ*B and Nrf2 signalling, reducing apoptosis.

**Figure 5:**
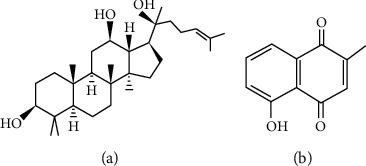
Chemical structures of saponins and quinones with pharmacological potential: (a) The saponins of ginsenosides; (b) the quinones of plumbagin.

Although saponins and quinones may be useful in the treatment of IDD, their effects have not been fully explored. More work is needed (Table 5 and Figure 5).

## 4. Mechanisms of Action of Natural Products Treating IDD

### 4.1. Natural Products Inhibit Inflammation

**Figure 6:**
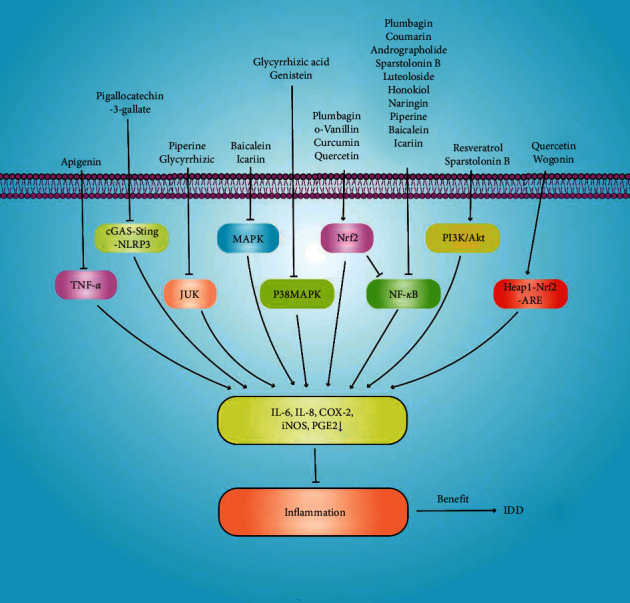
Natural products inhibit inflammation-related signalling pathways and reduce the expression of the inflammatory cytokines IL-6, IL-8, COX-2, iNOS, and PGE2, thereby delaying intervertebral disc degeneration.

Inflammation is a complex physiological response to cellular damage, infection, and tissue injury [152]. As intervertebral disc cells degenerate, the corresponding increased levels of secreted proinflammatory cytokines trigger IDD progression. TNF-*α*, IL-6, IL-8, IL-4, and PGE2 trigger the inflammatory cascade; they promote NP ECM degradation, cell senescence, and apoptosis [34]. Inflammation is a critical component of IDD. Natural products reduce the expression of IL-6, IL-8, COX-2, iNOS, and PGE2, thereby delaying IDD. Natural products affect different signalling pathways alone or in concert (Figure 6).

### 4.2. Natural Products Inhibit Oxidative Stress

**Figure 7:**
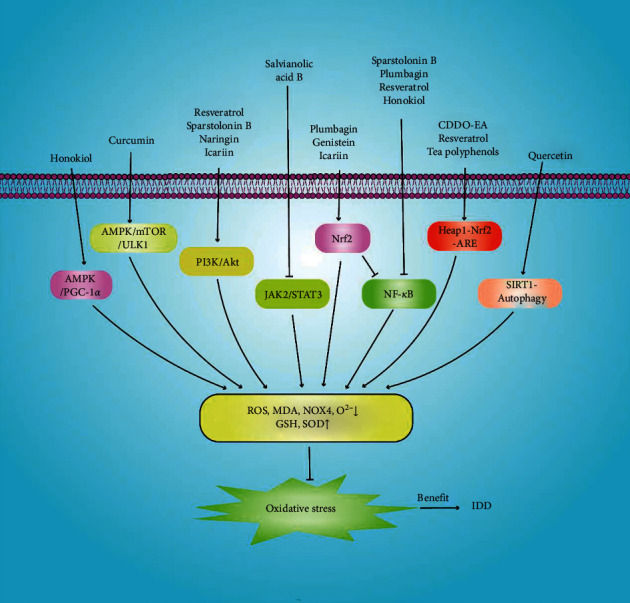
Natural products increase GSH and SOD expression and decrease those of ROS, MDA, NOX4, and O^2−^ by inhibiting the oxidative stress-related signalling pathways involved in intervertebral disc degeneration.

Oxidative stress is an imbalance between the levels of pro-oxidants and antioxidants; excess ROS cause cellular damage [153]. ROS are produced through the normal metabolism of mitochondria and peroxisomes, as well as by various cytoplasmic enzymes that either promote cell survival and tissue renewal or inhibit the expression of cell survival genes [154]. As IDD develops, ROS production by intervertebral discs increases [155]. Excessive ROS production disturbs intracellular redox homeostasis and triggers NP cell apoptosis and senescence, as well as ECM degradation. Therefore, oxidative stress is also important in terms of IDD. Natural products reduce oxidative stress and maintain NP cell redox homeostasis (Figure 7).

### 4.3. Natural Products Inhibit Apoptosis and ECM Degradation

**Figure 8:**
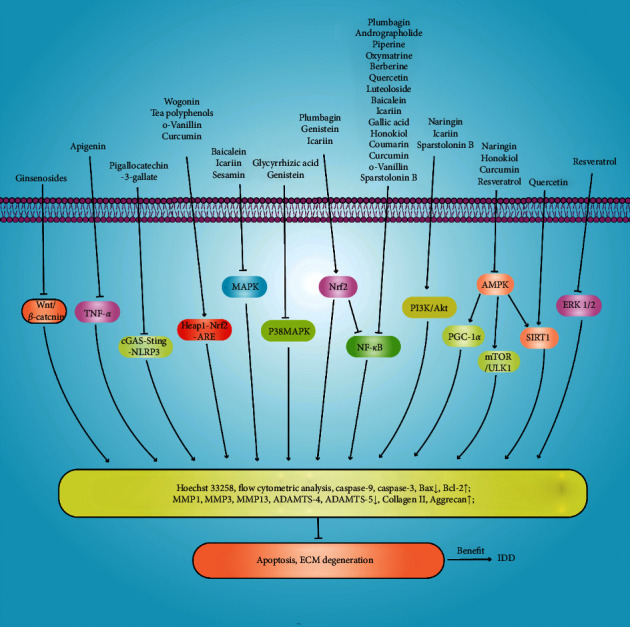
Natural products inhibit apoptosis and ECM degeneration through different mechanisms to prevent intervertebral disc degeneration.

Apoptosis is a form of programmed death controlled by genes that maintain homeostasis. Abnormal NP cell apoptosis plays an important role in IDD. Hoechst 33258 flow cytometric analysis is used to detect apoptosis. Natural products reduce the expression of the apoptotic proteins caspase-3, caspase-9, Bax, and Bcl-2. Collagen II and aggrecan are the principal components of the ECM, which structurally and biochemically supports intervertebral disc cells [156]. ECM degradation is a principal characteristic of IDD; MMPs and ADAMTS are the principal catabolic enzymes. Therefore, inhibition of apoptosis and matrix catabolism may slow IDD progression (Figure 8).

Inflammation and oxidative stress are important in terms of IDD development; the molecular mechanisms differ. NP cell apoptosis and ECM degeneration accelerate IDD; natural products inhibit NP cell apoptosis and ECM degeneration. Natural products affect NP cell apoptosis and senescence, as well as matrix anabolism, by inhibiting inflammation- and oxidative stress-related signalling, thus delaying IDD progression. Other factors (mitochondrial dysfunction and DNA damage) are also involved. More basic research is needed.

## 5. Clinical Applications

The drugs used to treat IDD relieve symptoms; they do not reverse IDD. Intervertebral disc tissue lacks blood vessels and nerves. Intervertebral disc structure and function are maintained by the osmotic effect of the CEP, reducing the efficacies of oral and intravenous drugs. There is an urgent need to develop effective drugs and reasonable routes of intervertebral disc administration. Nanoparticles have large surface areas and pore volumes, which allow them to effectively load drugs [157]. A combination of a nanomaterial and oxymatrine increased drug anti-IDD activity, suggesting a novel route of drug administration [132]. Natural products are prime sources of drug development [158]. Currently, most of the work on natural products for IDD has focused on preclinical nature. Although the natural product has not been studied in clinical practice, its powerful pharmacological activity has great potential in the treatment of IDD. The Sprague Dawley rat IDD model establishes an intervertebral disc injury in a tail vertebra via needle puncture. Natural products were given by gavage, intraperitoneal injection, and local injection. We summarise the progress of preclinical research in Table 6.

## 6. Conclusion and the Future

Oxidative stress and inflammation accelerate IDD. The intervertebral disc accumulates ROS; NP cells secrete inflammatory cytokines that activate IDD signalling pathways, compromise NP cell function, and trigger disc dysfunction and structural changes. Increased apoptosis and ECM degradation are also involved. Inhibition of oxidative stress, inflammation, apoptosis, and ECM degradation may slow IDD progression. Natural products can prevent and treat human diseases at low cost, as recognised in Asia and elsewhere. Natural products reduce ROS, MDA, and O^2−^production, lowering oxidative stress damage. Natural products inhibit the expression of the inflammatory cytokines IL-1*β*, TNF-*α*, COX-2, iNOS, PGE2, and IL-6, reducing injury to NP cells. Natural products inhibit apoptosis and regulate metabolism. Natural products have been shown (in preclinical studies) to slow IDD development. However, most studies regarding natural products are published in low-impact journals. The synergistic and multitargeting effects of natural products are not exhibited by synthetic drugs. A multitargeting multilevel approach may yield new IDD treatments; more work is needed. We hope that our review highlights the importance of natural products when seeking to treat and prevent IDD.

## Figures and Tables

**Table 1:** Natural phenolics with pharmacological potential.

Name	Source	Antioxidant stress	Anti-inflammatory	Antiapoptosis	Promote matrix anabolism	Promote proliferation	Signal path	Type of study	References
Resveratrol	Cinnamon bark, gourd, and polygonum cuspidatum	ROS, O^2−^, MDA↓			Collagen II, aggrecan↑, MMP3/13, ADAMTS-4↓	CCK-8↑	Nrf2/HO‐1	A	[18]
			IL-1*β*↓	Caspase-3, Bax↓, Bcl-2↑		CCK-8↑	PI3K/AKT	A	[44]
				Caspase-3/9, Bax↓, Bcl-2↑		CCK-8↑	ERK1/2	C	[46]
		ROS↓				CCK-8/EdU↑	ROS/NF-*κ*B	A	[47]
				Flow cytometric analysis↓	Collagen II↑, MMP13, ADAMTS-5↓	CCK-8↑	SIRT1/p21/p16	B	[48]
			TNF-*α*↓		MMP3↓	CCK-8↑	AMPK/SIRT1	B	[49]
					Collagen II, aggrecan↑, MMP1↓		SIRT1	B	[50, 51]
Coumarin	Solanaceae, Rutaceae, Umbelliferae		COX-2, iNOS, PGE2, TNF-*α*, IL-6↓		Collagen II, aggrecan↑, MMP3/13↓	CCK-8↑	NF-*κ*B	B	[19]
Sparstolonin B	Solanaceae	NOX2↓	TNF-*α*, IL-1*β*, IL-6↓	Caspase-3/9↓		CCK-8↑	NF-*κ*B or PI3K/AKT	A	[62]
Curcumin	Rhizome	SOD↑, ROS, MDA↓		Caspase-3/9, Bax↓, Bcl-2↑	Collagen II, aggrecan↑, MMP3/13, ADAMTS-4/5↓	CCK-8↑	AMPK/mTOR/ULK1	A	[68]
o-Vanillin	Metabolite of curcumin		IL-6, IL-8↓		Collagen II, aggrecan↑, MMP3/13↓		Nrf2 and NF-*κ*B	B	[69]
Honokiol	The leaves of Magnolia plants	ROS↓, SOD↑	COX-2, iNOS, IL-1*β*, IL-6↓	Caspase-3/9, Bax↓	Collagen II, SOX-9↑, MMP3/13, ADAMTS-4/5↓	CCK-8↑	NF-*κ*B or NLRP3	A	[74]
		SOD↑, ROS, MDA↓		Caspase-9↓		CCK-8↑	AMPK/PGC-1*α*/SIRT3	A	[76]
Salvianolic acid B	*Salvia miltiorrhiza*	ROS, MDA↓, GSH, SOD2↑		Caspase-3, Bax↓, Bcl-2↑		MTT↑	JAK2/STAT3	A	[79]
Tea polyphenols	Green tea	NOX4, ROS↓	iNOS↓		Collagen II, aggrecan SOX-9↑, MMP3↓	CCK-8↑	Keap1/Nrf2/ARE	B	[82]
Pigallocatechin-3-gallate	Green tea		IL-1*β*, TNF-*α*, IL-6, IL-10↓	Caspase-3/9, Bax↓, Bcl-2↑		CCK-8, migration↑	cGAS/Sting/NLRP3	B	[84]
Gallic acid	Grape seeds, Chinese rose			Flow cytometric analysis↓	ADAMTS-4↓	CCK-8↑	NF-*κ*B	B	[87]
Sesamin	Sesame seeds		IL-1*β*, TNF-*α*, iNOS, NO, COX-2, PGE2↓		Collagen II↑, MMP-1/3/13, ADAMTS-4/5↓		MAPK	A	[89]

A: rat model of NP in vitro, in vivo. B: human model of NP in vitro. C: pig model of NP in vitro.

**Table 2:** Natural flavonoids with pharmacological potential.

Name	Source	Antioxidant stress	Anti-inflammatory	Antiapoptosis	Promote matrix anabolism	Promote proliferation	Signal path	Type of study	References
Icariin	Epidemic	ROS↓		Caspase-3, Bax↓, Bcl-2↑, TUNEL↓		CCK-8↑	Nrf2	B	[93]
			COX-2, iNOS, PGE2↓		Collagen II, aggrecan↑, MMP3, ADAMTS-4↓	CCK-8↑	MAPK and NF-*κ*B	B	[94]
		ROS↓		Caspase-3, Bax↓, Bcl-2↑		CCK-8↑	PI3K/AKT	A	[95]
Naringin	Citrus fruits		TNF-*α*↓		Collagen II, aggrecan↑, MMP3↓	MTT↑		B	[100]
				Caspase-3, Bax↓, Bcl-2↑	Collagen II↑, MMP13↓	CCK-8↑	AMPK	A	[101]
			TNF-*α*, IL-6↓		Collagen II, aggrecan↑, MMP3/13, ADAMTS-4/5↓		NF-*κ*B	B	[102]
		ROS↓		Caspase-3, Bax↓, Bcl-2↑		CCK-8↑	PI3K/Akt	A	[103]
Genistein	Soybeans	ROS↓		Caspase-3, Bax↓, Bcl-2↑	Collagen II↑, ADAMTS-5, MMP13↓	CCK-8↑	Nrf2	A	[107]
			TNF-*α*, IL-1*β*↓		Collagen II, aggrecan↑, MMP3↓	CCK-8↑	p38 MAPK	A	[108]
Wogonin	*Scutellaria baicalensis* Georgi		COX-2, iNOS↓		Collagen II↑, MMP3/13, ADAMTS-4↓	CCK-8/EdU↑	Nrf2/ARE	A	[111]
Luteoloside	Honeysuckle, lettuce, silver flower		TNF-*α*, COX-2, iNOS, PGE2↓	Caspase-3, Bax↓, Bcl-2↑	Collagen II, aggrecan↑, MMP13, ADAMTS-5↓		NF-*κ*B	A	[114]
Quercetin	Vegetables, fruits, and teas	ROS↓		Caspase-3↓	Aggrecan↑, MMP13↓	CCK-8↑	SIRT1-autophagy	C	[117]
			IL-6, IL-8↓		MMP13, MMP3↓	EDU↑	Nrf2/NF-*κ*B	C	[118]
Baicalein	*Scutellaria baicalensis* and *Scutellaria lateriflora*		iNOS, COX-2, TNF-*α*, IL-6, PGE2, NO↓		Collagen II, aggrecan↑, MMP13, ADAMTS-5↓	CCK-8↑	NF-*κ*B and MAPK	A	[120]
Apigenin	Asteraceae		COX-2, IL-2, IL-6, IL-8, IL-17, IFN-*γ*, IL-1*β*↓		Aggrecan↑, MMP1/3/9, ADAMTS-4/5↓	CCK-8↑		A	[122]
Kaempferol	*Ginkgo* and *Moringa*		IL-6↓		Collagen II, aggrecan, SOX-9↑, MMP3/13↓	CCK-8↑	NF-*κ*B	D	[124]

A: rat model of NP in vitro, in vivo. B: human model of NP in vitro. C: human model of NP in vitro and rat model of IDD in vivo. D: rat model of BMSCs in vitro.

**Table 3:** Natural alkaloids with pharmacological potential.

Name	Source	Antioxidant stress	Anti-inflammatory	Antiapoptosis	Promote matrix anabolism	Promote proliferation	Signal path	Type of study	References
Berberine	Rhizoma coptidis			Caspase-3, Bax↓, Bcl-2↑	Collagen II, aggrecan↑, MMP3/13, ADAMTS-4/5↓	CCK-8↑	NF-*κ*B	B	[127]
		ROS↓		Caspase-3, Bax↓, Bcl-2↑		CCK-8↑	IRE1/JNK	A	[128]
				Caspase-3, Bax↓, Bcl-2↑	Collagen II, aggrecan↑, MMP13, ADAMTS-5↓		Autophagy	A	[129]
Oxymatrine	*Sophora flavescens*		IL-6↓	Hoechst 33258, flow cytometric analysis↓	Collagen II, aggrecan↑, MMP2/3/9/13↓	CCK-8↑	NF-*κ*B	A	[131]
			IL-6↓	Hoechst 33258, flow cytometric analysis↓	Collagen II↑, MMP3/9↓			A	[132]
Piperine	Black pepper		IL-1*β*, TNF-*α*, IL-6, iNOS↓		Collagen II, aggrecan↑, MMP3/13, ADAMTS-4/5↓	CCK-8↑	NF-*κ*B and MAPK	A	[135]

A: rat model of NP in vitro, in vivo. B: human model of NP in vitro.

**Table 4:** Natural terpenoids with pharmacological potential.

Name	Source	Antioxidant stress	Anti-inflammatory	Antiapoptosis	Promote matrix anabolism	Promote proliferation	Signal path	Type of study	References
Andrographolide	*Andrographis paniculata*		COX-2, PGE2↓	Caspase-3↓	Collagen II, aggrecan↑, MMP3/13, ADAMTS-4/5↓	CCK-8↑	NF-*κ*B	B	[138]
				Caspase-3, Bax↓, Bcl-2↑	MMP3/9/13↓		NF-*κ*B	B	[139]
Glycyrrhizin	Licorice		TNF-*α*, IL-6, IL-8, iNOS↓	Caspase-3/8↓	Collagen II↑	CCK-8↑	p38/p-JUK	B	[141]
CDDO-ethyl amide	Oleanolic acid	ROS↓		Flow cytometric analysis↓		CCK-8↑	MAPK and Nrf2	A	[142]

A: rat model of NP in vitro, in vivo. B: human model of NP in vitro.

**Table 5:** Natural saponins and quinones with pharmacological potential.

Name	Source	Antioxidant stress	Anti-inflammatory	Antiapoptosis	Promote matrix anabolism	Promote proliferation	Signal path	Type of study	References
Ginsenosides	Ginseng genus			Flow cytometric analysis↓	Collagen II, aggrecan↑	CCK-8↑, cyclin D1↓	Wnt/*β*-catenin	A	[148]
		ROS↓	TNF-*α*↓	Caspase-3, Bax↓, Bcl-2↑	Collagen II, aggrecan↑, MMP3, ADAMTS-5↓	CCK-8/EdU↑	NF-*κ*B	B	[149]
Plumbagin	Graphite	ROS↓, GSH, SOD2↑	TNF-*α*, IL-1*β*, IL-6↓	Caspase-3/9↓		MTT	NF-*κ*B and Nrf2	A	[151]

A: rat model of NP in vitro. B: human model of NP in vitro.

**Table 6:** Natural products used for preclinical research.

Name	Intervention approach	Drug dosage	Duration (weeks)	Summary of main findings (compared with IDD group)	References
Resveratrol	Intragastric administration	50 mg/kg/d	4	Decrease Pfirrmann MRI grade; Alcian blue: increase the content of ECM; SA-*β*-Gal staining: the number of positive cells decreases	[18]
Curcumin	Injected intraperitoneally	100 mg/kg/d	4	Decrease Pfirrmann MRI grade; HE: IVD morphology improvement; immunohistochemistry: collagen II and aggrecan content increased	[68]
Honokiol	Injected intraperitoneally	30 mg/kg/d	4	Decrease Pfirrmann MRI grade; HE and Alcian blue: IVD morphology improved; immunohistochemistry: MMP13 and ADAMATS-5 expression decreased	[74]
	Intragastric administration	40 mg/kg/d	4	Decrease Pfirrmann MRI grade; HE: IVD morphology improvement; immunofluorescence: upregulation of SIRT3 expression	[76]
Salvianolic acid B	Intragastric administration	20 mg/kg/d	6	Decrease Pfirrmann MRI grade; HE, safranin-O fast green, and Alcian blue: IVD shape improvement	[79]
Tea polyphenol	Local injection	100 *μ*M/week	4	Decrease Pfirrmann MRI grade; HE, safranin-O fast green, and Alcian blue: IVD shape improvement; immunohistochemistry: collagen II and aggrecan content increased	[82]
Icariin	Injected intraperitoneally	30 mg/kg/d	8	Decrease the Pfirrmann MRI grade; increase the height of the IVD; immunohistochemistry: aggrecan content increased; TUNEL: decreased rate of positive cells	[93]
Naringin	Injected intraperitoneally	80 mg/kg/d	4 and 12	Decrease Pfirrmann MRI grade; HE: IVD morphology improved	[101]
Genistein	Intragastric administration	100 mg/kg/d	4	X-ray: increased intervertebral space height; HE: improved IVD morphology; Immunofluorescence: upregulated expression of Nrf2	[107]
	Local injection	5 *μ*g/mL, 10 *μ*g/mL, 20 *μ*g/mL/week	2 and 4	Decrease Pfirrmann MRI grade; HE: improve the structure of the intervertebral disc in a dose-dependent manner; immunohistochemistry: increase the expression of collagen II in a dose-dependent manner	[108]
Wogonin	Local injection	50 *μ*M/week	4 and 8	Decrease Pfirrmann MRI grade; HE: IVD morphology improvement	[111]
Luteoloside	Injected intraperitoneally	10 mg/kg/d	4 and 8	Decrease the Pfirrmann MRI grade; X-ray: increased intervertebral space height; HE and safranin-O fast green: IVD morphology improvement	[114]
Quercetin	Injected intraperitoneally	100 mg/kg/d	8	X-ray: increased intervertebral space height; HE and safranin-O fast green: IVD morphology improvement; immunofluorescence: downregulation of caspase-3 expression, upregulation of SIRT1 expression	[117]
Berberine	Injected intraperitoneally	150 mg/kg/d	8	Decrease Pfirrmann MRI grade; HE and safranin-O fast green: IVD morphology improvement; TUNEL: decreased rate of positive cells	[128]
	Intragastric administration	150 mg/kg/d	4	Decrease Pfirrmann MRI grade; HE: IVD morphology improvement; immunofluorescence: downregulation of caspase-3 expression	[129]
Oxymatrine	Local injection	Oxymatrine 10 *μ*M; Oxymatrine 10 *μ*M + LIP100 *μ*g/*μ*L	4	X-ray radiology: oxymatrine-LIP can partially restore the height of the IVD	[132]

## Data Availability

No data were used to support this study.
